# Draft Genome Sequence of *Salmonella enterica* Serovar Typhi Strain STH2370

**DOI:** 10.1128/genomeA.00104-14

**Published:** 2014-02-20

**Authors:** Camila Valenzuela, Juan A. Ugalde, Guido C. Mora, Sergio Álvarez, Inés Contreras, Carlos A. Santiviago

**Affiliations:** a Departamento de Bioquímica y Biología Molecular, Facultad de Ciencias Químicas y Farmacéuticas, Universidad de Chile, Santiago, Chile; b Marine Biology Research Division, Scripps Institution of Oceanography, University of California, San Diego, La Jolla, California, USA; c Centro de Genómica y Bioinformática, Facultad de Ciencias, Universidad Mayor, Santiago, Chile; d Unidad de Microbiología, Facultad de Medicina, Universidad Andres Bello, Santiago, Chile

## Abstract

We report the draft genome sequence of *Salmonella enterica* serovar Typhi strain STH2370, isolated from a typhoid fever patient in Santiago, Chile. This clinical isolate has been used as the reference wild-type strain in numerous studies conducted in our laboratories during the last 15 years.

## GENOME ANNOUNCEMENT

*Salmonella enterica* serovar Typhi is a human-restricted pathogen causing typhoid fever, a severe systemic disease with an estimated incidence of ~22 million cases per year worldwide (1, 2). Our group has been interested in understanding the molecular aspects of *S.* Typhi pathogenicity for more than 30 years. Initial studies were conducted using the reference strain Ty2 (3, 4). However, this strain is attenuated *in vivo* and *in vitro* partly due to a frameshift mutation within the *rpoS* gene (5), which makes it less suitable for virulence assays. On the other hand, although reference strain *S.* Typhi CT18 remains fully virulent, it presents resistance to multiple antibiotics (6), making it almost impossible to perform genetic assays using resistance to common antibiotics as a selectable marker. Therefore, we decided to use an antibiotic-sensitive virulent strain to continue our studies. We chose *S.* Typhi STH2370, a clinical strain isolated in 1991 from a female typhoid fever patient admitted at the Hospital de Enfermedades Infecciosas Dr. Lucio Córdova in Santiago, Chile. Since then, this clinical isolate has been used as the reference wild-type strain in several studies conducted in our laboratories to date (see examples in references 7–20).

To further characterize strain STH2370, we decided to sequence its entire genome. To do that, total DNA was extracted using the GenElute bacterial genomic DNA kit (Sigma-Aldrich) from bacteria grown overnight in LB at 37°C with agitation. The DNA was used to prepare a library with the Nextera DNA sample preparation kit (Illumina). High-throughput sequencing of the library was performed on the MiSeq platform (Illumina) with a 2 × 250-bp paired-end run using MiSeq reagent kit version 2 (500 cycles). This sequencing strategy produced 7,449,129 read pairs for a total of 3.2 Gbp and an estimated coverage of 650×. The reads were analyzed and checked for quality using FastQC (http://www.bioinformatics.babraham.ac.uk/projects/fastqc/). Adapters and quality trimming were performed using Nesoni (http://www.vicbioinformatics.com/software.nesoni.shtml). When possible, read pairs from the same fragments were combined using FLASH (21). Genome assembly was performed using SPAdes (22) and resulted in 54 contigs (N_50_, 206,306 bp; mean length, 85,894 bp; maximum length, 420,648 bp) that were aligned to the genome of *S.* Typhi reference strain CT18 using Mauve (23, 24), manually curated, and processed for automatic annotation using the NCBI Prokaryotic Genome Annotation Pipeline (http://www.ncbi.nlm.nih.gov/genome/annotation_prok/).

Our results indicate that the chromosome of strain STH2370 is composed of approximately 4,802,151 bp, with an average G+C content of 52.1% and a total of 4,406 coding sequences. No plasmid sequences were found in the sequencing data. A brief analysis of the STH2370 genome reveals several differences with respect to strain CT18, including the presence of a P4-like phage remnant (~14 kb) near the ortholog of gene STYt047, a phage (~36.5 kb) located between genes *btuC* and *himA* (and related to one located at the same position in the genome of *Escherichia coli* strain SE11), the replacement of SPI-10 (ΦST46) by another P4-related phage (~12 kb), the absence of phage ST18, and the lack of IS*1* elements.

### Nucleotide sequence accession numbers.

The draft genome sequence of *S. enterica* serovar Typhi strain STH2370 has been deposited at DDBJ/EMBL/GenBank under the accession no. JABZ00000000. The version described in this paper is version JABZ01000000.
